# Funding patterns for biomedical research and infectious diseases burden in Gabon

**DOI:** 10.1186/s12889-021-12201-w

**Published:** 2021-11-24

**Authors:** Olouyomi Scherif Adegnika, Yabo Josiane Honkpehedji, Fabrice Mougeni Lotola, Selidji Todagbe Agnandji, Ayola Akim Adegnika, Bertrand Lell, Elisa Sicuri

**Affiliations:** 1 Centre de Recherches Médicales de Lambaréné, Lambaréné, Gabon; 2 Institute for Tropical Medicine, University of Tübingen, Tübingen, Germany; 3 Department of Parasitology, Leiden University Medical Center, Leiden, the Netherlands; 4 German Center for Infection Research (DZIF), African partner institution, CERMEL, Lambaréné, Gabon; 5 German Center for Infection Research (DZIF), partner site Tübingen, Tübingen, Germany; 6 Department of Medicine I, Division of Infectious Diseases and Tropical Medicines, Medical University of Vienna, Vienna, Austria; 7 ISGlobal, Hospital Clinic, Universitat de Barcelona, Barcelona, Spain; 8 Health Economics Group, Department of Infectious Disease Epidemiology, School of Public Health, Imperial College London, London, UK

**Keywords:** Gabon, Research financing, Infectious diseases, Research partnership

## Abstract

**Background:**

Biomedical research plays an important role in improving health. There seems to exist a negative correlation between the amount of biomedical research funding and disease burden from all Sub-Saharan African countries. In this study, we describe funding patterns for biomedical research, explore the correlation between funding and burden of diseases, and quantify inequalities in funds distribution across diseases in Gabon over the period 2005–2015.

**Methods:**

Data on medical research funds from 2005 to 2015 were retrieved through a structured questionnaire distributed to Gabonese biomedical research institutions and by consulting online databases. Data on the burden of diseases were gathered from the World Health Organization and the Institute for Health Metrics and Evaluation. We used Kendall rank correlation coefficient to explore the correlation between cumulative funds over time and the burden of disease. The inequality distribution of funding across diseases was assessed through Gini coefficient and Lorenz curve.

**Results:**

Biomedical research funding was characterized by a remarkable growth from 2005 to 2010 and a decline from 2010 to 2014. Funds were mostly from external sources and from partnerships. There was inequality in research funds allocation across diseases and malaria was far the most funded disease. There was a significant negative correlation between cumulative funding and the burden of HIV, tuberculosis, and of Helminthiasis (from 2006 to 2010) suggesting that research may be contributing to the management of such diseases. A positive, although not significant, correlation was found between cumulative funds and malaria burden.

**Conclusions:**

The negative correlation between HIV and tuberculosis cumulative funding and burden suggests that research may be contributing to the management of such diseases but further research is needed to assess the causal direction of such as relationship. As the burden of non-communicable diseases is increasing, more research funds should be focused on those. While research partnerships have been and will remain fundamental, Gabon should increase the amount of national funds to overcome periods of reduced research funding flows from abroad.

**Supplementary Information:**

The online version contains supplementary material available at 10.1186/s12889-021-12201-w.

## Background

Since independence, countries of sub-Saharan Africa (SSA) have made significant progress on the healthcare front allowing universal health coverage (UHC) to be set as a goal in the national health strategies [1, 2]. Although UHC has not yet been widely achieved, some countries are making substantial progress. This is the case of South Africa, Cabo Verde, Kenya and Namibia among others [3, 4]. As a result of the efforts made, many health indicators have improved over the last decades. In terms of disease specific indicators, the global number of malaria cases and of malaria-associated deaths, mostly still occurring in SSA, declined by 30 and 37%, respectively, between 2005 and 2015 [5]. The number of deaths due to Human Immunodeficiency Virus infection and acquired immune deficiency syndrome (HIV/AIDS) and tuberculosis (TB), also largely concentrated in SSA, globally dropped by 30 and 17% respectively, over the same time period [5].

Despite such encouraging improvement, the all-cause morbidity and mortality burden in SSA is still unacceptably high while at halfway toward completion of the epidemiological transition, characterized by (a) the double presence of infectious and non-communicable diseases [6], and (b) a low level of satisfaction of citizens with their health and with health care services, suggesting that wide gaps still exist towards a satisfactory improvement of the health system and relative health improvements [7]. This warns health policy makers that there is still a lot to do on the ground of UHC which is key to the improvement of population health. To reach UHC, more investments for strengthening health systems are necessary and, particularly, stable health system funding is needed: this entails reforming not only the health system but the whole socio-economic and financial system [8].

In the past decade, concerns about the disproportionate burden of disease and mortality in SSA have arisen. The health-gap in SSA could be improved with investments in healthcare delivery but also by boosting research [9]. There is a dearth of research and development of products, tools, and strategies for diseases that are specific to low-income countries and to SSA in particular. For example, most of the so-called “neglected tropical diseases” (NTDs) are not yet vaccine preventable and are still treated with old drugs characterized by low efficacy levels and a high incidence of adverse effects [10]. When innovative health products are available, new and context specific strategies are needed to make them accessible to the population at risk [11]. In response to this, many donors and research agencies have increased the funding mainly for biomedical research but also for socio-economic and implementation research in the form of cross-cutting themes, and mostly through fostering cross-continental and country partnerships [12]. The substantial increase in funding for research has led to either the foundation of new or the enhancement of already existing medical research centers across SSA [9]. This has largely contributed to the discovery (e.g., the first candidate malaria vaccine), and scaling-up of new preventions and treatments for HIV/AIDS, tuberculosis, and malaria (e.g., the expansion of antiretroviral treatment for HIV positive individuals) [9]. Importantly, research investment in SSA also lays the foundations for boosting capacity building of African scientists [13, 14].

Channeling funding to support biomedical and basic research may be seen not as a priority [9]. In fact, several low- and middle-income African countries are experiencing difficult economic situations and some are still characterized by the presence of large pockets of extreme poverty. Although it is not easy to assess the impact of health-related research, some attempts not specific to SSA have been made and final outcomes of such assessments have been identified. These include broader socio-economic indicators, stressing the fact that health research also has a positive impact in the fight against poverty [15]. What can certainly be said for SSA is that the large investment in research has effectively translated into enhanced research capacities, and research is finally carried out at large scale across the region. For example, according to the 2020 annual report of The European & Developing Countries Clinical Trials Partnership (EDCTP), over the period 2014–2020, through its second program, 40 sub-Saharan African countries through 252 African institutions participated in EDCTP projects, and have been granted 59% of its total grant value [16]. According to that report, the recruitment sites for collaborative clinical studies funded by EDCTP were hosted by 36 sub-Saharan African countries and 25 sub-Saharan African countries were awarded a total of 196 fellowships for the career development of African researchers [16].

Health research is likely to play an important role in freeing resources for the health system. In a recent study, Jaffar et al. [17] concluded that altogether the financial benefits of health research exceeded the costs of research through improved care outcome, although the quantification of the financial benefits to the health system is challenging. In general, it has been recognized that it is hard to identify the association between the amount of research funds and impact on health, due to uncertainty, complexity of the biological mechanisms, of the immune responses, and to the actual access of the population in need to health technologies [18].

In such a complexity, several factors need to be considered by agencies seeking to fund biomedical research. Fixing research priorities should take into account among other factors, the disease burden in order for research investments to impact the health of populations [19]. Research investments are currently unevenly distributed across geographical areas and pathologies. Despite showing a similar large burden of disease, a few SSA countries receive several large grants for research activities, whilst other countries rely only on scarce funding [20]. Head et al. [21] found that Tanzania, Uganda, Kenya, Malawi, and Ghana were the top five SSA countries to receive the greatest funding amount for malaria research, and 18 nations received more than US$10 million of research funding, and that at the same time, some countries, eight in total (Botswana, Cape Verde, Central African Republic, Chad, Congo Brazzaville, Djibouti, Mauritania, and Sierra Leone) were not allocated research investments at all. In summary, both Head et al. [21] and Herrick and Reades [22] showed low level investments in Central Africa, with resources mostly concentrated in the western and eastern sides of the region. Scarce resources invested in Central Africa may be due to a combination of factors such as a lack of infrastructure, poor governance, weakness of scientific human resources, and a particularly complex political and conflictual situation. For example, according to the 2018 Ibrahim Index of African governance (IIAG)^1^ which measures and monitors governance performance in African countries (iiag.online), the score of the Central Africa region is 39.4, while the one of West Africa region is 54.3, and the one of East Africa is 44.8. The score ranges from 0, the worst, to 100, the best possible score.

Inequities also exist across pathologies with many diseases receiving little funding, including those that are particularly problematic in Africa, such as the NTDs (e.g., Buruli ulcer, schistosomiasis, and human African trypanosomiasis) [20, 23]. On the contrary, the coverage of HIV/AIDS, in terms of research conducted and funding allocated is relatively high, followed by malaria and TB [23]. Some recent studies have found a strong relationship between the funding for biomedical research and the burden of disease [24–26] while other studies have concluded that some diseases are disproportionately funded relative to their burden [24, 25, 27]. Notably, Barrenho et al. [28] focused on the direction of the relationship between disease burden and research. They used drug innovation as an indicator of health research and assessed the existence of a mismatch between disease burden and pharmaceutical innovation. Specifically, they found that for some high burden disease categories (i.e., cardiovascular and circulatory diseases), innovation is disproportionately concentrated in diseases with high disease burden and large market size, whereas for others, including Neglected Tropical Diseases, innovation is disproportionately concentrated in low burden diseases. For example, they found that more drugs have been launched for dengue than for rabies while more DALYs are associated with the former rather than the latter.

In terms of sources, research funds for SSA countries come mainly from foreign donors. In their study, Head et al. [21] found that the largest investment for malaria research was provided by the US National Institutes of Health ($292.0 million, representing 36% of the total funding), followed by the Bill & Melinda Gates Foundation with a total investment of $144.1 million (18%). The study also found that investment funding for some countries came mainly from one donor namely the EDCTP [21].

To summarize, most funding for scientific research, training and infrastructure upgrade in Africa comes from high-income countries, which build, in some cases, on already existing infrastructure [9, 29]. As a further characterization of research funding, according to the Global Forum, 59% of health research funds for low- and middle-income countries in 2003 came from the public sector, 32% from the private for-profit sector (mainly pharmaceutical companies), and 9% from the private not-for-profit sector [30].

Our study is linked to the literature on health research funding decisions, which aims to set an ethical and moral framework for the allocation of research funds [31]. According to this literature, medical research should be chosen according to its social value. While the social value of interventions depends on the capacity to improve equity [32], often there are difficulties and uncertainties even to identify a priori the relationship between health research and health outcomes. With this in mind, we describe funding patterns for all main infectious diseases with a specific focus on Gabon, Central Africa. We also explore the correlation between funding amount and the burden of several diseases and quantify potential inequalities in the distribution of funds across diseases. While we only look at correlations with no causality concerns, we expect diseases with higher cumulative funds over time showing lower burden of disease. We believe that this information may constitute a very first step towards the generation of relevant information for health research decision making. Only a few studies have been conducted in settings other than Central Africa to analyze the funding of biomedical research and its links with the disease burden. A broad picture is missing on the funding for biomedical research in sub-Saharan Africa, especially in Gabon, considering which are the main funded diseases and the relationship with the disease burden. Head et al. [21] presented evidence on funding for biomedical research from all SSA countries including Gabon, but on malaria only, and found correlations between temporal levels of investment and disease burden.

### The Gabonese research, health and health system context

Situated in Central Africa, Gabon is an upper/middle-income country with a population estimated in 2018 by the World Bank to be about 2,200,000 inhabitants, and a large geographic area relatively to its small population [33].

In comparison with the remainder Central Africa countries, Gabon has experienced a remarkable biomedical research expansion. This is likely to be due to the leadership of the few research institutions present in the country and the capacities of their actors to attract funding. The political stability, the ease of carrying out research due to the acceptance of research activities by the political community, the commitment of stakeholders to medical research activities, and the existence of a minimum of infrastructure could be further reasons. The funds mobilized by the few Gabonese research institutions increase their capacities, which, in turn, increase the attractiveness of the country for conducting research, forming a virtuous circle.

While medical research centers based in Gabon sometimes conduct single-center research projects, most projects are conducted in partnerships with other institutions based in other countries, both from the South and the North. Partnerships are established through consortia that involve several research institutions for the conduct of collaborative research projects, memoranda of understanding for long-term partnerships, and the development of research networks for the exchange and sharing of knowledge and skills. A network that includes research institutions based in Gabon is the Central Africa Network on Tuberculosis, HIV/AIDS, and Malaria (CANTAM) which is funded by EDCTP, a partnership of which Gabon is also a member.

In a study describing the patterns of biomedical science production in a sub-Saharan research center conducted in Lambaréné, Gabon, Agnandji et al. [34] found that transnational studies were the most important share of research activities, with European and/or North American partners coordinating the scientific, technical, and financial aspects of the projects. In addition to transnational research, the research center also conducts academic or ancillary research, where the design, implementation, analysis and preparation of publication are all carried out within the research center [34]. However, the interaction between the locally designed and transnational studies was not specified, nor was their funding [34].

In the World Report, Collins et al. [20] found that Gabon, over the period 2012–2016, received funding for biomedical research from 5 funding organizations: EDCTP (19 records, 41%), National Institute of Health-NIH (16 records, 35%), European Commission-EC (8 records, 17%), Institute National de la Santé et de la Recherche Médicale - INSERM (2 records, 4%) and Medical Research Council-MRC (1 record, 2%).

The Gabonese health system is organized into three sectors namely the public sector which includes the civil and military sectors, the para-public sector and the private sector. The civil public sector has three levels, namely the peripheral level which is that of the operationalization of primary care, the intermediate level which plays a role of technical support to regional and departmental structures, and the central level which plays a role of strategic orientation, normative and regulator of the whole system. The epidemiological situation of Gabon is characterized by transmissible infectious and parasitic diseases, non-communicable diseases, emerging and re-emerging diseases, injuries from road accidents, alcoholism, smoking, drug addiction, occupational diseases and those food-related, sexually transmitted infections including HIV / AIDS, and sickle cell disease. Health research is poorly structured in Gabon. The country does not have a structured medical research agenda and research is carried out by very few institutions including public medical schools, para-public institutions and private institutions.

Biomedical research is funded and conducted with the aim of generating new knowledge, studying the causes and potential treatments and prevention of diseases in order to contribute to the improvement of population health. It is therefore important to analyze the link between the funding allocated to biomedical research and the population health through the burden of disease.

## Methods

### Research setting

This study took place in Gabon from 2016 to 2018. The study was organized from Centre de Recherches Médicales de Lambaréné (CERMEL), an independent research institution in Lambaréné, Gabon, focusing on research on malaria, multidrug-resistant tuberculosis, and worm infections. Initially a Research Laboratory as part of the Albert Schweitzer Foundation, the center has successively changed its name first to Medical Research Unit (MRU) in 2001 with a financial and administrative independence from the Albert Schweitzer Hospital, then to CERMEL in 2011, its current name, with a new legal status. But the research center still shares the same physical address with the hospital. CERMEL is now a leading research center in the Central African sub-region which has established partnerships with several research institutions and academics around the world.

### Data collection

To collect data for this study, we used a structured questionnaire to gather financial data from all health research institutions in Gabon that provided information from their project administration offices. We searched data for the longest possible period, and could obtain them for the period from 2005 to 2015. The questionnaire included questions on the characteristics of the institution, funding amounts and funding sources during the studied period (external, internal, etc.), funding by disease (malaria, HIV, tuberculosis, etc.), funding by activity type (prevention, treatment, other), funding by research type (clinical trial, capacity building, epidemiology, immunology), and funding by funder type (public sector, private sector, philanthropic sector). The questionnaire is included in supplementary material (See Additional file 1). In addition to the data gathered from the questionnaire, we also extracted data on biomedical research funding for Gabon from funders websites or funding database (e.g. G-Finder, and World RePORT maintained by the US National institute of Health, which are online open-access, databases highlighting biomedical research funding from some of the world’s largest funding organizations), and did web searches in Google, Google Scholar, and PubMed using the terms “Gabon”, “Funding”, “Financing”, “Research”, “biomedical”, “medical”, and “health” to search for biomedical research awards focusing on Gabon.

The data on the burden of those diseases that received a positive amount of funds, were extracted from the World Health Organization (WHO) and the Institute for Health Metrics and Evaluation (IHME) web sites.

### Data analysis

We have combined the data collected from research institutions and those extracted from databases, and eliminated duplicates. We did not consider in-kind resources. Only funding resulting in transfer of money from the donor to the beneficiary was taken into account. Each extracted award from the web was linked to the disease it focused on and put in the following categories: treatment, prevention, basic research, and applied research. When the funding was common to two or more diseases, the funding amount was split into the number of diseases and equally attributed to each disease. Research funds that were specific to ethics or social science were classified as “cross-cutting” and diseases that were funded only within one project were classified as “other”. Where applicable, the grant funds were converted to Euro using the average exchange rate in the year of the award. We performed the conversion using the “Historical Currency Converter” tool available on the website of OANDA (www1.oanda.com).

We used Disability-Adjusted Life Years (DALYs) for Gabon from the IHME’s global burden of diseases results tool as a measure of disease burden. We considered the DALYs caused by the diseases for which we have funding data, that is, for the diseases that resulted having a positive level of associated funds. We used DALYs as a measure for disease burden as research funding should help with the improvement of at least one of the DALYs component, that is, morbidity or mortality.

In order to establish whether there are possible links between funding and disease burden, and to measure the strength of this connection if applicable, the correlation between the cumulative funding amounts and the burden (DALYs) of the main diseases present in Gabon, over time was explored through the Kendall’s test. The correlation was quantified through the Kendall rank correlation coefficient. To measure the inequality in the allocation of research funding across diseases, we plotted the Lorenz curve of the cumulative funding amount for biomedical research relatively to the disease burden where diseases were ordered from highest burden to lowest, and calculated the Gini coefficient.

We used the statistical software R version 3.5.1 to analyze the data [35].

## Results

**Fig. 1:**
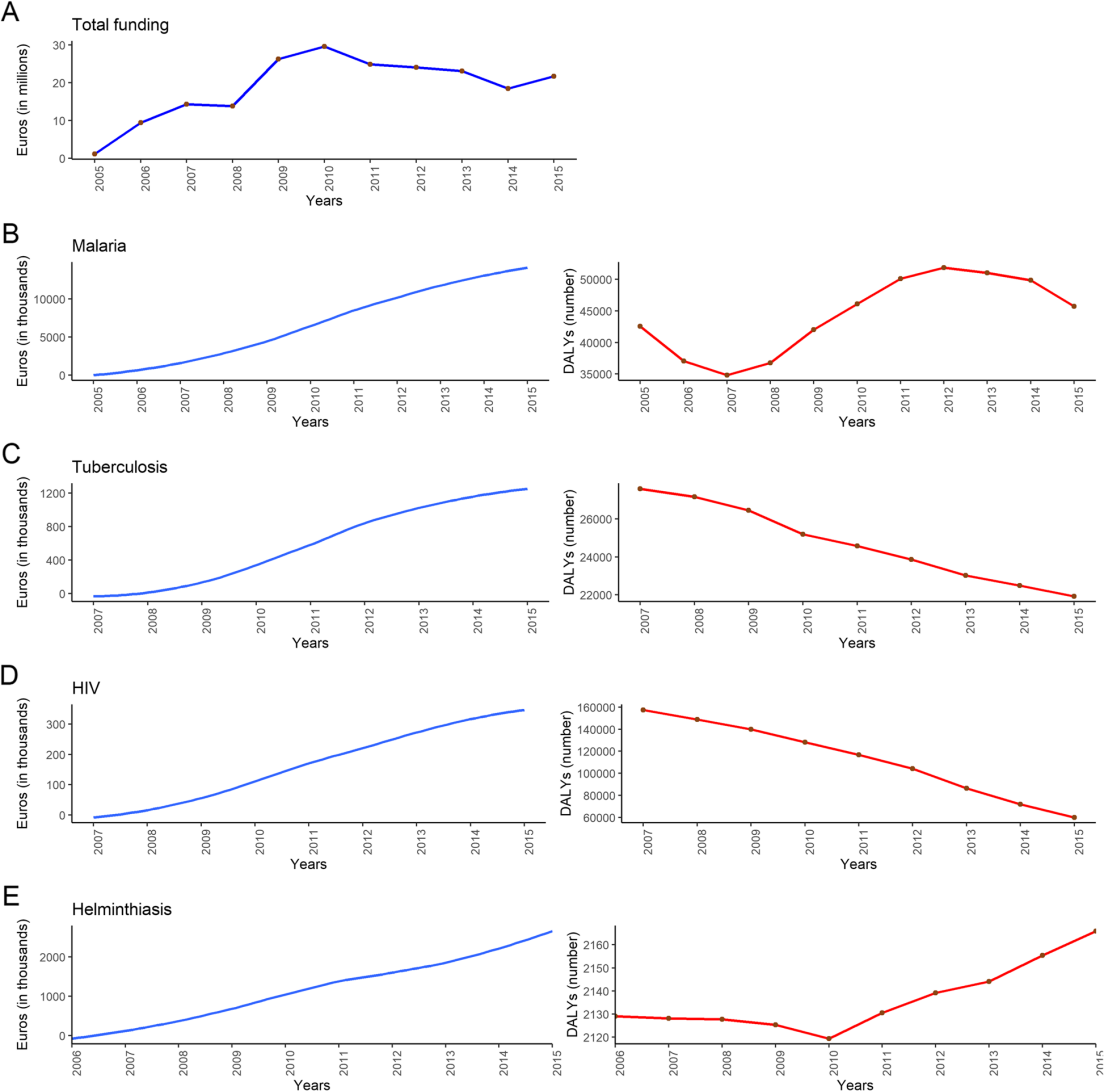
Trends for total funding (panel **A**) over time in Gabon. Panel **B**, **C**, **D** and **E** show cumulative funding (left hand side) and disease burden (right hand side) over time for specific diseases

A total of 74 biomedical research funded projects were gathered from 5 research institutions in Gabon. The 74 biomedical research projects were funded for a total of 20,6 million euros over the period 2005–2015, with a median yearly funding amount of 2165 thousand euros (range: 112 to 2960 thousand euros). By far, most of the funds came from external sources and were granted by international funders, with 58% of the total funding amount coming from the public sector, 37% from the philanthropic sector, and 5% from the private sector. The annual funding for biomedical research in Gabon for the period 2005–2015 contains two main phases: a phase of growth which extends from 2005 to 2010 and a decline phase running from 2010 to 2014. During the phase of decline, the funding amounts for biomedical research in Gabon decreased successively from year to year before slightly recovering in 2015 (Fig. 1A). By disease, malaria was by far the most funded in Gabon. Forty (52% of research project focusing on a disease) disease-related biomedical research funded projects with a total funding amount of 14,3 million euros (70%) focused on malaria. In terms of funding amount, malaria is followed respectively by helminthiasis, tuberculosis, and viral diseases (Table 1).

**Table 1:** Funding for biomedical research projects by disease in Gabon between 2005 and 2015

Disease	DALYs (Number)	No of funded projects	Total funding amount	Yearly median (IQR)
Malaria	487,949	40 (51.9%)	14,309,712 € (70.2%)	99,000 € (39000–317,000)
Helminthiasis	23,481	12 (15.6%)	2,737,087 € (13.4%)	170,000 € (16000–333,000)
TB	278,479	8 (10.4%)	1,278,284 € (6.3%)	83,000 € (51000–224,000)
Viral diseases^a^	1272	2 (2.6%)	1,257,413 € (6.2%)	629,000 € (434000–823,000)
HIV	1,343,268	6 (7.8%)	342,661 € (1.7%)	70,000 € (23000–86,000)
Bacterial diseases^b^		4 (5.2%)	327,935 € (1.6%)	55,000 € (8000–129,000)
Other^c^	71,953	5 (6.5%)	128,866 € (0.6%)	7000 € (6000–53,000)
TOTAL	2,206,402	68 (100%)	20,381,960 € (100%)	

^a^ Dengue and Ebola hemorrhagic fever

^b^ *Helicobacter pylori*, Streptococcus, and *S. aureus*

^c^ Food allergy, fever without source (FWS), hematology, chronic obstructive pulmonary disease (COPD), and hemosporidic parasites and zoonotic viruses

By activity type, prevention received the greatest funding amount. It was funded a total amount of 10,4 million euros (51% of the total funding amount) for 12 projects (16%) while treatment was funded 5,2 million euros (25% of the total funding amount) for 14 projects (19%). Other activity types (‘descriptive’) which are neither prevention nor treatment and corresponding to research areas such as social science, ethics, immunology, or epidemiology was funded 5,0 million euros (24% of the total funding amount) through 48 (65%) projects.

By research type, clinical trials were allocated 15,6 million euros (76%) for 26 (35%) funded projects; capacity building was funded with 1,7 million euros (8%) for 13 (18%) funded projects, epidemiology was allocated 1,6 million euros (8%) for 22 (30%) projects, and immunology received a total funding amount of 1,5 million euros (7%) for 7 (9%) projects. Altogether, the top four research types together received 99% of the total funding amount through 92% of the number of funded biomedical research projects. The remaining research types (‘ethics’, ‘nested in clinical trial’ and ‘social science’) received 1% of the total funding amount. By cross-cutting research, ethics and social science have equally shared the number of funded projects, 3 (50%) each. In terms of funding amount, ethics has been funded a total amount of 143,8 thousand euros (55% of the total cross-cutting research funding), and the social science has been funded a total amount of 119,6 thousand euros (45% of the total funding amount for cross-cutting research).

The Kendall rank coefficient (tau) highlighted a strong negative correlation between cumulative funding amount for HIV and relative burden of disease (tau = − 0.98, *p* < 0.01), between cumulative funding amount for tuberculosis and relative burden of disease (tau = − 0.99, *p* < 0.01), and between cumulative funding amount for helminthiasis and relative disease burden for the period from 2006 to 2010 (tau = − 0.96, *p* = 0.017). A strong positive but not significant correlation was found between cumulative funding amount for malaria research and relative burden of disease (tau = 0.81, *p* = 0.06), and a strong positive and significant correlation was found between cumulative funding amount for helminthiasis and relative disease burden for the period from 2010 to 2015 (tau = 0.99, *p* < 0.01).

**Fig. 2:**
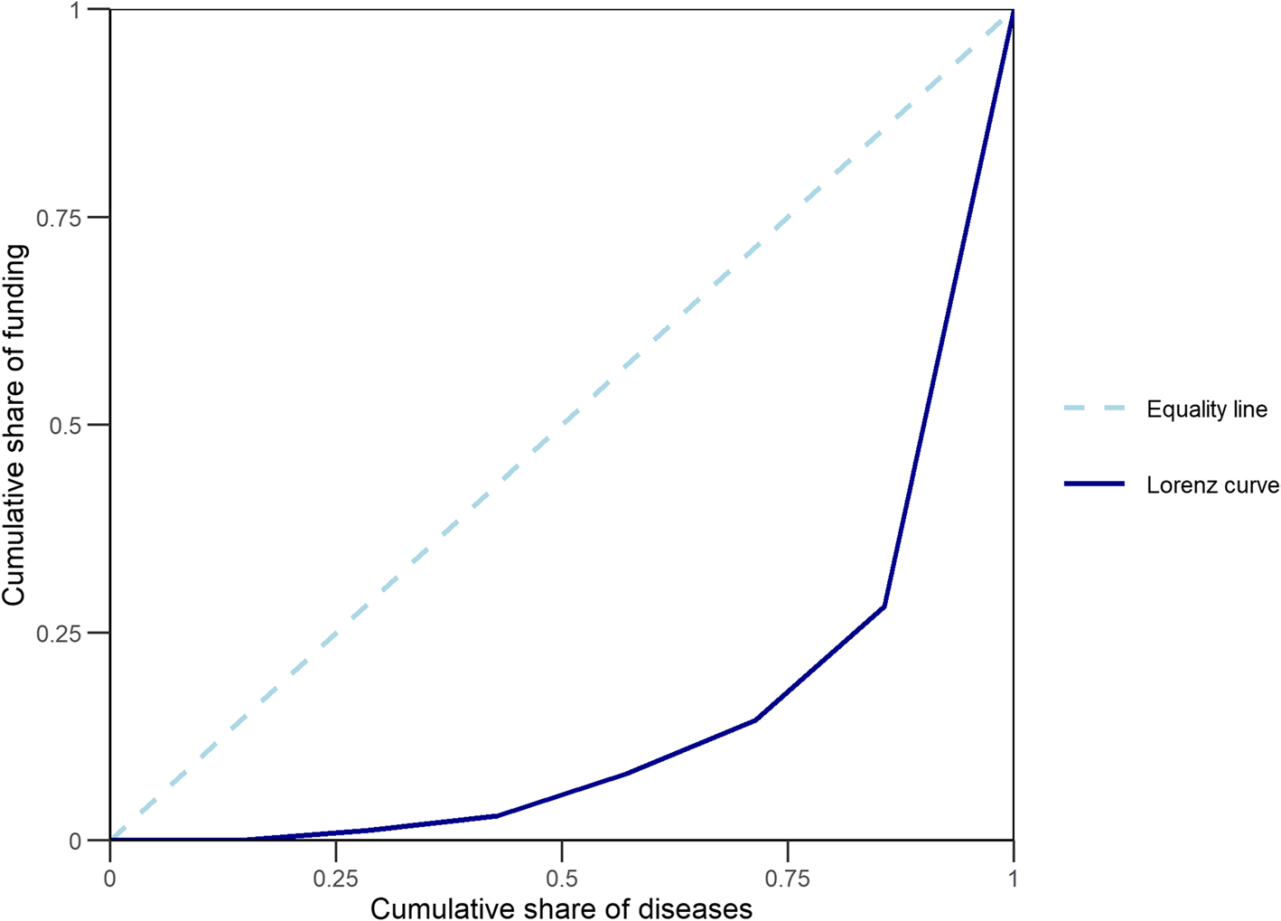
Lorenz curve for funding distribution across diseases in Gabon from 2005 to 2015. Diseases are ordered from highest to lowest burden on the x-axis

In the context of this study, the Gini coefficient is 0.65; which suggests a strong inequality in the distribution of research funding across diseases. In Fig. 2, the Lorenz curve represents the cumulative share of funding amount for biomedical research on the vertical axis relative to the cumulative share of the burden of disease represented by the disability-adjusted life years (DALYs) on the horizontal axis. The curve tends to the extreme right of total inequality; for example, 80% of the diseases with highest burden received less than 30% of funding. This suggests inequity in the distribution of funds.

In terms of disease burden, HIV has the highest associated DALYs (1,3 million), followed by malaria (487,9 thousand), tuberculosis (278,5 thousand), helminthiasis (23,5 thousand), and viral diseases (1,3 thousand).

The cumulative funding amounts for biomedical research on malaria in Gabon grew over the studied period while the DALYs caused by malaria decreased between 2005 and 2007, increased from 2007 to 2012, and decreased between 2012 and 2015 (Fig. 1B).

The cumulative funding amounts for tuberculosis grew from 2007 to 2015 while the DALYs caused by tuberculosis decreased during the same period (Fig. 1C).

While the cumulative funding amounts for HIV grew from 2007 to 2015, HIV DALYs decreased during the same period (Fig. 1D).

As regard to helminthiasis, its cumulative funding amount was characterized by a growth between 2006 and 2015, while its DALYs decreased from 2006 to 2010 and increased from 2010 to 2015 (Fig. 1E).

## Discussion

This study has described the funding for biomedical research in Gabon for the period from 2005 to 2015, and showed that a total of 20.6 million euros were injected into biomedical research. Funds experienced a sharp increase from 2005 to 2010 and a decrease from 2010 to 2014 followed by a partial recovery in 2015.

Our analysis permits the drawing of a clear outline of the biomedical research carried out in Gabon over the period evaluated: the largest share of funds (70% of the total) is devoted to malaria: funds are mainly awarded by public international donors; are mainly for clinical trials and for prevention.

Importantly, the association between the cumulative amount of funds and burden of disease has been found negative and significant for tuberculosis, HIV, and also for helminthiasis for the period from 2006 to 2010. Although this is only an association, it may be interpreted in the sense that the accumulation of funds for tuberculosis, HIV, and helminthiasis is contributing to a reduction of disease burden. Interestingly, Head et al. [21] found a negative correlation between temporal levels of investment and malaria disease burden. On the contrary, we found a positive although not significant correlation. This difference can be explained by the fluctuating malaria burden over years in Gabon, which does not reflect the global decreasing trends. In general, the funded diseases (malaria, helminthiasis, tuberculosis, HIV and viral diseases) during the studied period reflect the epidemiological situation of the country, where infectious diseases are still very prevalent.

HIV has the highest associated DALYs during the studied period. As HIV prevalence is not particularly high in Gabon (3,8% in 2018 according to the World Bank), this might be explained by the fact that HIV is a chronic and life-long disease and that increases disability in comparison with the remaining diseases. Notably, in our analysis we undertook only a bivariate analysis between funding and disease burden; we are aware that a diverse set of factors can be explanatory of the disease burden (technology, disease control, health system, weather conditions, climate, etc.) in addition to health research. However, in our analysis, we wanted to focus the attention on disease burden and funding only, over time, while assuming everything else constant.

The sharp funds increase over the period 2005–2010, the decrease from 2010 to 2014 before increasing again between 2014 and 2015 may be explained by the ensuing reasons. Beginning of the years 2000 were characterized by an increase in research funding for Global Health and the increasing pattern in Gabon from 2005, from a negligible amount recorded in the same year 2005, mirrors such a global pattern [36]. As biomedical research in Gabon during the studied period was mainly funded by international donors, the funding decline could be due to the financial crisis of 2008–2009 in the US and 2 years later in Europe. The final resumption could be explained by the Ebola outbreak that occurred in 2014 and to which the international community reacted by the urgent financing of research on this highly deadly viral disease.

A remarkable feature of the research funds Gabon has been receiving is that almost all of the projects are the result of North / South collaborations and the funds come from the North only. Thus, the lack of South / South partnerships and of locally led projects arises [37]. Southern biomedical research institutions, alone or in partnership, should be put in a condition to develop projects, and coordinate such projects with or even without North research institutions as partners.

The top 3 funders were respectively Bill & Melinda Gates Foundation, European and Developing Countries Clinical Trial Partnership (EDCTP), and the European Commission (EC). They together provided almost 75% of total biomedical research funding, and malaria alone received about 70% of the total funding. This shows the interest the funders place on malaria research, which remains one of the leading causes of mortality and morbidity in developing countries although not the main one in many contexts, including Gabon [38, 39].

Our findings are different from the ones of Head et al. [21] where it was found that EDCTP provided 79% of the research investment on malaria in Gabon and was the main funder. This difference is potentially due to the fact that some of the data considered in the present study are likely not to have been included in the study of Head et al. This could be the case, for example, of some data held by research institutions that have not been published on the websites of funding agencies or which do not appear in dedicated databases. This information was, instead, captured by our national survey.

Globally, according to data from G-Finder, most of the funding for biomedical research comes from the public sector, and this has been confirmed in this study.

Currently, most of funding for biomedical research in Gabon is directed at communicable diseases. There is a total lack of research on non-communicable disease while they are becoming very prevalent [40] .

It seems there is a lack of disease burden arbitration for funds allocation which goes more to communicable diseases where researchers in Gabon have more capacity. Such allocation of funds is understandable given that diseases, such as malaria, are endemic in SSA, including Gabon, and not in high-income countries, where most of research on non-communicable diseases is carried out.

For the welfare of the SSA population, it would be important that the disease burden is taken into account in the funding allocation for biomedical research [41]. To achieve this, capacity strengthening of medical researchers in the field of non-communicable disease will be crucial.

Biomedical research could impact health care and contribute to the improvement of population health. To achieve this, it is key for research findings to be implemented and for the population to have access to research outputs, such as vaccines or new treatments; however, this is difficult without implementation research and health system research. The research in Gabon appears to be very vertical, i.e., focused on the disease rather than on the health system. Therefore, in order for biomedical research to contribute fully to improving the population health in Gabon, it is essential that health systems and implementation research are conducted at a larger scale [42].

For example, biomedical research studies could be designed in a way to include a component of implementation research. An example of implementation research embedded in biomedical studies is the recent Ebola vaccine study where the clinical trial was designed in order to take into account the implementation of a fast response in the case of a new outbreak [43].

In Gabon, a concrete example where biomedical research has contributed to improving the health of the population is the study of the combination of artesunate/amodiaquine which has been conducted in Gabon and in other countries. Indeed, following the initial registration for the manufacture of the drug, Gabon is one of the first endemic countries to have granted authorization for its local marketing, and on the recommendation of WHO to use the combination in malaria control programs and for first-line treatment for African children with uncomplicated malaria, Gabon has made it a national policy and artesunate/amodiaquine is thus the first-line treatment for uncomplicated malaria [44, 45].

The lack of a national policy, of a research agenda, and of a funding plan for medical research in Gabon did not allow us to analyze the current funding in relation to forecasted needs, and this leads us to formulate the following recommendations: a) a national biomedical research agenda should be put in place with an adequate funding plan, in accordance with the priorities of the health system, to establish rules that ease and strengthen medical research, and increase and diversify the capacity of researchers in the biomedical field; b) the establishment of a dialogue between research funders, researchers and decision-makers to help better allocate resources; c) more research on the causal impact of health research on health outcomes as well as on equity and on equality issues should be fostered; d).

more effort, from all the stakeholders, should be placed on health data collection and gathering as national routine surveillance for all the diseases and health concerns. Our recommendations are theoretical and do not consider issues of resources constraints; however, we believe that these are necessary steps to take to enhance the effectiveness of health research towards improving health.

Our study is not without limitations. One of these is the validity of the data used for disease burden. In fact, we used DALYs data for Gabon published by the IHME which are generally estimates derived from models; these estimates may contain missing data and may be slightly different from DALYs calculated with actual data.

Another limitation is that in addition to online consultation of funders databases, we asked research institutions for their financial information to extract what would not be available in the funders databases. The failure of research institutions to provide us with the exhaustiveness of their financial information constitutes missing data which could change the results of the study.

## Conclusions

This study shows that biomedical research in Gabon is mainly funded by external resources. Inequality exists in the allocation of funding across diseases and malaria, a major disease in Africa, including Gabon, accounts for most of the funding for biomedical research activities for the period 2005–2015, while it is not the disease that causes the highest disease burden. While the negative correlation between tuberculosis, HIV, and helminthiasis cumulative funding and burden of diseases are apparent, suggesting that research may be contributing to the management of such diseases, further research would be needed to establish causality. As the burden of non-communicable diseases is increasing in Gabon, as for all SSA, more research funds should be focused on those. Gabon should also increase the amount of national funds towards research for overcoming periods of reduced funding flows from abroad.

## Supplementary Information

**Additional file 1.** Questionnaire on data related to biomedical research funding in Gabon. This is the questionnaire used to collect data on medical research funds from Gabonese biomedical research institutions.

## Data Availability

The datasets used and/or analysed during the current study are available from the corresponding author on reasonable request.

## Abbreviations

CERMEL: Centre de Recherches Médicales de Lambaréné
DALY: Disability-Adjusted Life Years
EC: European Commission
EDCTP: European & Developing Countries Clinical Trials Partnership
HIV: Human Immunodeficiency Virus
HIV/AIDS: Human Immunodeficiency Virus infection and Acquired Immune Deficiency Syndrome
IHME: Institute for Health Metrics and Evaluation (IHME)
IIAG: Ibrahim Index of African Governance
NTD: Neglected Tropical Diseases
SSA: Sub-Saharan Africa
TB: Tuberculosis
UHC: Universal Health Coverage
WHO: World Health Organization

## Footnotes

1 The IIAG is published since 2007 and it assesses the governance performance of 54 African countries over the most recent 10-year period. It provides a framework and dashboard to assess the provision of public goods and services and the results of public policies in African countries. The IIAG provides specific scores and trends at continental, regional and national levels in Africa, across various thematic dimensions of governance such as security, justice, rights, economic opportunities, and health.
