# Supplementary material for: Funding patterns for biomedical research and infectious diseases burden in Gabon
Source: BMC Public Health. 2021 Nov 24;21:2155. doi: 10.1186/s12889-021-12201-w (PMC8611934; doi:10.1186/s12889-021-12201-w)
Supplement: Supplementary file 1 — Additional file 1. Questionnaire on data related to biomedical research funding in Gabon. This is the questionnaire used to collect data on medical research funds from Gabonese biomedical research institutions. [file 12889_2021_12201_MOESM1_ESM.pdf]

Funding patterns for biomedical research in Gabon: an example of research partnership in global health and an exploration of infectious diseases burden

Questionnaire to health research institutions in Gabon

Questionnaire on data related to biomedical research funding in Gabon.

This survey is carried out within the framework of our study which explores the funding patterns for biomedical research and infectious disease burden in Gabon. The questionnaire is designed to collect health research financial data from biomedical research institutions.

We guarantee the security and confidentiality of your data and assure you they will appear only in the aggregated results of our research work.

The questionnaire includes six sections as follows:

I. Characteristics of the institution;
II. *Funding amount and sources;*
III. Funding by research topic;
IV. Funding by activity type
V. Funding by research type;
VI. Funding by funder type.

Please, add as many pages, columns, and lines as you would need to answer this questionnaire.

I. Characteristics of the institution

Name of the institution:

Year of creation:

Sector the institution belongs to:
☐ Public ☐ Private non for-profit ☐ Para public ☐ Private for-profit ☐ Other

Type of institution:
☐ Government agency ☐ Hospital ☐ Health center ☐ University/Health school
☐ Private biomedical research institution ☐ Other

Primary function:
☐ Biomedical research ☐ Healthcare ☐ Academy ☐ Other

II. Total funding amount and sources

Monetary Unit:

NB: The amounts must be expressed in the same monetary unit.

Year	Internal	External	Other (Specify)	Total
Total				

III. Funding by research topic

Monetary Unit:

NB: The amounts must be expressed in the same monetary unit.

Year	Malaria	Tuberculosis	HIV/AIDS	Helminths	Dengue	Ebola	Other (Specify)
Total							

IV. Funding by activity type

Monetary Unit:

NB: The amounts must be expressed in the same monetary unit.

Year	Prevention	Treatment	Social science	Ethics	Other (Specify)
Total					

V. Funding by research types

Monetary Unit:

NB: The amounts must be expressed in the same monetary unit.

Year	Clinical trial	Epidemiology	Immunology	Capacity building	Nested to clinical trial	Other (Specify)
Total						

VI. Funding by funder type

Monetary Unit:

NB: The amounts must be expressed in the same monetary unit.

Year	Public sector	Private sector	philanthropic sector	Other (Specify)
Total				
